# Efficacy and safety of endoscopy-specific dual-channel supraglottic airways for upper gastrointestinal endoscopic and transesophageal instrumentation procedures: a systematic review and meta-analysis

**DOI:** 10.3389/fmed.2026.1879284

**Published:** 2026-07-17

**Authors:** Yanqin Mi, Zhengfang Qi, Yan Wei, Yatao Liu, Zhaohui Gao

**Affiliations:** 1 The First School of Clinical Medicine of Lanzhou University, Lanzhou, China; 2 Department of Anesthesiology and Operation, First Hospital of Lanzhou University, Lanzhou, China

**Keywords:** airway management, dual-channel supraglottic airway, endoscopic retrograde cholangiopancreatography, hypoxemia, upper gastrointestinal endoscopy

## Abstract

**Background:**

Airway management during upper gastrointestinal endoscopic procedures and other transesophageal instrumentation remains challenging because the airway and procedural device share the upper aerodigestive tract. Endoscopy-specific dual-channel supraglottic airways (dc-SGAs), including the LMA^®^ Gastro™ Airway and the Jcerity Endoscoper™ Airway, are designed to maintain ventilation while permitting passage of an endoscope or transesophageal probe through a separate procedural channel. Nevertheless, their comparative efficacy and safety remain uncertain across heterogeneous procedures and comparator strategies.

**Methods:**

We conducted a systematic review and meta-analysis of randomized controlled trials comparing endoscopy-specific dc-SGAs with endotracheal tubes, other placed airway or access devices, or non-invasive oxygen therapy during upper gastrointestinal endoscopic or transesophageal instrumentation procedures. Observational studies were summarized narratively but were not included in pooled analyses.

**Results:**

Fifteen studies were included, including 13 randomized controlled trials involving 2,481 participants in the quantitative synthesis and 2 observational studies summarized narratively. Endoscopy-specific dc-SGAs showed comparable first-attempt airway device and endoscope/probe insertion success to comparator strategies and shortened airway device insertion time. Effects on endoscope/probe insertion time and endoscopist satisfaction were inconsistent and varied across comparator types. dc-SGAs reduced intraoperative hypoxemia, mainly in comparisons with non-invasive oxygen therapy. Postoperative sore throat showed a comparator-dependent pattern, with fewer events compared with endotracheal tubes but more events compared with non-invasive oxygen therapy.

**Conclusion:**

Endoscopy-specific dc-SGAs may represent a feasible intermediate airway strategy for selected patients undergoing upper gastrointestinal endoscopic and transesophageal instrumentation procedures. They may improve airway placement efficiency and, compared with non-invasive oxygen therapy, reduce hypoxemia; however, these potential benefits should be interpreted in light of comparator-dependent throat discomfort, procedure heterogeneity, and the low-to-moderate certainty of evidence.

**Systematic review registration:**

https://www.crd.york.ac.uk/PROSPERO/view/CRD420251182546, identifier [CRD420251182546].

## 1 Introduction

Endoscopic retrograde cholangiopancreatography (ERCP), other upper gastrointestinal (GI) endoscopic procedures, and transesophageal instrumentation procedures are increasingly performed in patients with significant comorbidities and limited physiological reserves. Airway management in this setting is challenging because these procedures may be lengthy, performed in prone, semi-prone, lateral, or supine positions, and conducted outside the operating room. Deep sedation or monitored anesthesia care without a secured airway is common practice, but is associated with clinically relevant rates of hypoventilation, hypoxemia, and sedation-related adverse events, prompting ongoing debate over the optimal airway strategy for procedures involving upper aerodigestive tract instrumentation (1–3).

Current approaches to airway management during upper GI endoscopic and transesophageal instrumentation procedures range from low-flow nasal cannula or other non-invasive oxygen therapy to supraglottic airway devices and endotracheal intubation. Each option entails trade-offs between airway protection, gas exchange, procedural access, and resource use. Nasal cannula oxygen preserves spontaneous breathing but does not reliably prevent hypoxemia in high-risk or obese patients, especially under deep sedation. In contrast, endotracheal intubation offers more definitive airway control but requires general anesthesia, may require neuromuscular blockade, and entails more extensive equipment and staffing; therefore, it may be unnecessary for many selected cases (4, 5).

To address this gap, dual-channel supraglottic airways have been developed to facilitate airway management during procedures involving upper aerodigestive tract instrumentation. In this review, dual-channel supraglottic airways, hereafter referred to as endoscopy-specific dc-SGAs, refer exclusively to the LMA^®^ Gastro™ Airway and the Jcerity Endoscoper Airway, both of which provide a separate procedural channel in addition to a dedicated airway channel. These devices are designed to allow simultaneous ventilation and endoscope or transesophageal probe passage, while separating the airway and procedural channels (6–8). Early clinical studies suggest that endoscopy-specific dc-SGAs are feasible and may help maintain oxygenation or provide acceptable procedural conditions compared with non-invasive oxygen therapy or selected airway-device comparators, while representing a less invasive option than endotracheal intubation in selected patients (7, 9, 10).

Despite growing interest in endoscopy-specific dc-SGAs, current evidence remains fragmented and device choice is largely guided by local experience rather than robust comparative data. Most available studies are single-centre trials or observational cohorts with limited sample sizes, heterogeneous comparators, and variable outcome definitions. Moreover, available evidence spans different procedural settings, including ERCP, other upper GI endoscopic procedures, endoscopic variceal ligation, mixed therapeutic endoscopy, and transesophageal echocardiography. Although these procedures are not clinically identical, they share the common challenge of upper aerodigestive tract instrumentation competing with airway management. Clarifying the relative benefits and risks of endoscopy-specific dc-SGAs in this context has important clinical implications. If supported by comparative evidence, dc-SGAs may provide acceptable oxygenation and procedural conditions compared with endotracheal intubation while reducing invasiveness and facilitating recovery in selected patients. Compared with standard non-invasive oxygen therapy, dc-SGAs may also represent a more proactive yet still less invasive approach to airway management in selected patients undergoing upper GI endoscopic or transesophageal instrumentation procedures.

On this basis, we conducted a systematic review and meta-analysis to compare endoscopy-specific dc-SGAs with endotracheal tubes, other placed airway or access devices, and non-invasive oxygen therapy during upper GI endoscopic or transesophageal instrumentation procedures. We evaluated efficacy outcomes, including airway and endoscope/probe insertion performance, intraoperative oxygenation, and recovery time, as well as safety and provider-reported outcomes, including postoperative sore throat, hoarseness, blood staining or airway trauma, and endoscopist satisfaction. We aimed to assess the comparative efficacy and safety of dc-SGAs across different comparator strategies and procedural settings.

## 2 Methods

### 2.1 Study design and reporting

This systematic review and meta-analysis was conducted in accordance with the Preferred Reporting Items for Systematic Reviews and Meta-Analyses (PRISMA) 2020 statement (11). The quantitative synthesis focused on randomized controlled trials comparing endoscopy-specific dual-channel supraglottic airways (dc-SGAs) with alternative airway or oxygenation strategies during upper gastrointestinal endoscopic or transesophageal instrumentation procedures. Additional observational studies evaluating these devices were summarized narratively when they provided relevant feasibility or safety data, but were not included in the pooled effect estimates. The protocol was prospectively registered in the PROSPERO Registry (CRD420251182546). The completed PRISMA 2020 checklist is provided in the Supplementary material.

### 2.2 Eligibility criteria

Studies were eligible for inclusion if they met the following criteria:

(i) Population: Patients undergoing upper gastrointestinal endoscopic or transesophageal instrumentation procedures, including ERCP, diagnostic or therapeutic upper GI endoscopy, endoscopic variceal ligation, mixed upper GI endoscopic procedures, or transesophageal echocardiography.

(ii) Intervention: Use of an endoscopy-specific dual-channel supraglottic airway, defined as either the LMA^®^ Gastro™ Airway or the Jcerity Endoscoper™ Airway. These devices were considered eligible because they provide a dedicated airway channel and a separate procedural channel allowing passage of an endoscope or transesophageal probe.

(iii) Comparators: Eligible comparator strategies included:

a) endotracheal tubes;

b) other placed airway or access devices, such as single-channel supraglottic airways, gastrolaryngeal tubes, or endoscopy masks; and/or

c) non-invasive oxygen therapy, such as nasal cannula oxygen or nasal prongs.

(iv) Outcomes: Studies were eligible if they reported at least one clinically relevant efficacy, safety, or provider-reported outcome, including:

a) airway insertion performance, such as first-attempt success or insertion time;

b) endoscope or transesophageal probe insertion performance, such as first-attempt success or insertion time;

c) intraoperative oxygenation or respiratory adverse events, including hypoxemia or desaturation;

d) recovery-related outcomes, including emergence time, PACU time, or time to discharge readiness;

e) provider-reported procedural outcomes, such as endoscopist or anesthesiologist satisfaction; and/or

f) airway-related adverse events, including postoperative sore throat, sore throat scores, hoarseness, dysphagia, blood staining on the device, mucosal trauma, laryngospasm, aspiration, airway conversion, or procedure interruption.

(v) Study design: Randomized controlled trials were eligible for the quantitative synthesis. Comparative observational studies and clinically relevant non-comparative observational studies were summarized narratively when they provided feasibility or safety data for endoscopy-specific dc-SGAs, but were not included in pooled effect estimates.

We excluded case reports, small case series without sufficient outcome data, conference abstracts without sufficient extractable data, non-clinical studies, cadaveric or manikin studies, studies evaluating airway devices other than endoscopy-specific dc-SGAs, and studies not reporting any predefined outcome of interest. When multiple publications originated from the same or overlapping cohort, the report with the most complete and relevant data was used.

### 2.3 Search strategy

A systematic literature search was performed in PubMed, Embase, the Cochrane Library, and Web of Science from database inception to June 2026. The search strategy combined device-related terms, procedure-related terms, and airway-management terms. Device-related terms included “LMA Gastro”, “LMA Gastro Airway”, “Laryngeal Mask Airway Gastro”, “Gastro LMA”, “Jcerity Endoscoper”, “Jcerity Endoscoper Airway”, “Endoscoper Airway”, “dual-channel supraglottic airway”, “dual-channel laryngeal mask”, “dual-lumen supraglottic airway”, and “endoscopy-specific supraglottic airway”. Procedure-related terms included “ERCP”, “endoscopic retrograde cholangiopancreatography”, “upper gastrointestinal endoscopy”, “upper GI endoscopy”, “gastroscopy”, “esophagogastroduodenoscopy”, “oesophagogastroduodenoscopy”, “endoscopic variceal ligation”, “transesophageal echocardiography”, “transoesophageal echocardiography”, and “TEE”. The search strategy was adapted for each database using a combination of controlled vocabulary terms and free-text terms. Reference lists of included studies and relevant reviews were manually screened to identify additional eligible studies. No language restrictions were applied during the electronic search. Full-text articles were assessed when sufficient information was available for eligibility assessment and data extraction. The complete search strategies for each database are provided in Supplementary Table 1.

### 2.4 Study selection

Two reviewers independently screened titles and abstracts to identify potentially relevant studies. Full texts of candidate articles were then retrieved and assessed against the eligibility criteria. Discrepancies were resolved by discussion, with involvement of a third reviewer if necessary. The study selection process was documented using a PRISMA flow diagram.

### 2.5 Data extraction

Two reviewers independently extracted study characteristics, patient populations, procedure types, intervention devices, comparator strategies, anesthetic techniques, and reported outcomes using a standardized form. Comparator strategies were recorded as reported and categorized as endotracheal tube, other airway/access devices, or non-invasive oxygen therapy for subgroup interpretation. Extracted outcomes included airway device and endoscope/probe insertion performance, intraoperative hypoxemia, endoscopist satisfaction or procedural ease, postoperative sore throat (POST), recovery-related outcomes, and airway-related adverse events. For POST, incidence data were extracted preferentially; when multiple postoperative time points were reported, the earliest assessment was used for the primary analysis. Details of POST assessment methods and time frames are provided in Supplementary Table 2. When necessary, corresponding authors were contacted for clarification or missing information. If only medians and interquartile ranges were reported, they were approximated to means and standard deviations using established methods, where appropriate. If data values were represented in a graphical format, numerical data were extracted from graphs by WebPlot Digitizer (12).

### 2.6 Risk of bias assessment

Risk of bias in randomized controlled trials was assessed independently by two reviewers using the revised Cochrane Risk of Bias tool for randomized trials (RoB 2). Assessments were performed at the outcome level and considered five domains: bias arising from the randomization process, deviations from intended interventions, missing outcome data, measurement of the outcome, and selection of the reported result. Each domain and the overall risk of bias were judged as low risk, some concerns, or high risk. Because observational studies were summarized narratively and were not included in pooled effect estimates, they were appraised separately for major methodological limitations.

The certainty of evidence for each major outcome and comparator category was assessed using the Grading of Recommendations Assessment, Development, and Evaluation (GRADE) approach, considering risk of bias, inconsistency, indirectness, imprecision, and publication bias. The certainty of evidence was rated as high, moderate, low, or very low. Disagreements were resolved by discussion, with involvement of a third reviewer when consensus could not be reached.

### 2.7 Statistical analysis

Meta-analyses were performed using Review Manager version 5.4.1 when at least two randomized controlled trials reported comparable outcomes. Continuous outcomes were analyzed as mean differences (MDs) or standardized mean differences (SMDs) with 95% confidence intervals (CIs); medians with interquartile ranges or ranges were converted to means and standard deviations using the method of Hozo et al. (13). Dichotomous outcomes were pooled as risk ratios (RRs) with 95% CIs using the Mantel–Haenszel method (14). Random-effects models were used for the primary analyses. Heterogeneity was assessed using the χ^2^ test and *I*^2^ statistic (15).

Because comparator strategies differed in airway invasiveness and physiological function, subgroup analyses were performed according to comparator category when data permitted: endotracheal tube, other airway/access devices, and non-invasive oxygen therapy. Procedure-specific subgroup analyses, such as ERCP versus non-ERCP procedures, were not performed because of limited study numbers, mixed-procedure studies, and confounding between procedure type, device type, and comparator strategy. A two-sided *P*-value < 0.05 was considered statistically significant.

## 3 Results

### 3.1 Results of literature search

The study selection process is shown in the PRISMA flow diagram (Supplementary Figure 1). Ultimately, 15 studies met the eligibility criteria and were included in the systematic review, including 13 randomized controlled trials (RCTs) and 2 observational studies. The 13 RCTs were included in the quantitative synthesis, whereas the 2 observational studies were summarized narratively as supportive feasibility or safety evidence and were not included in the pooled effect estimates (7, 8, 10, 16–26).

**FIGURE 1:**
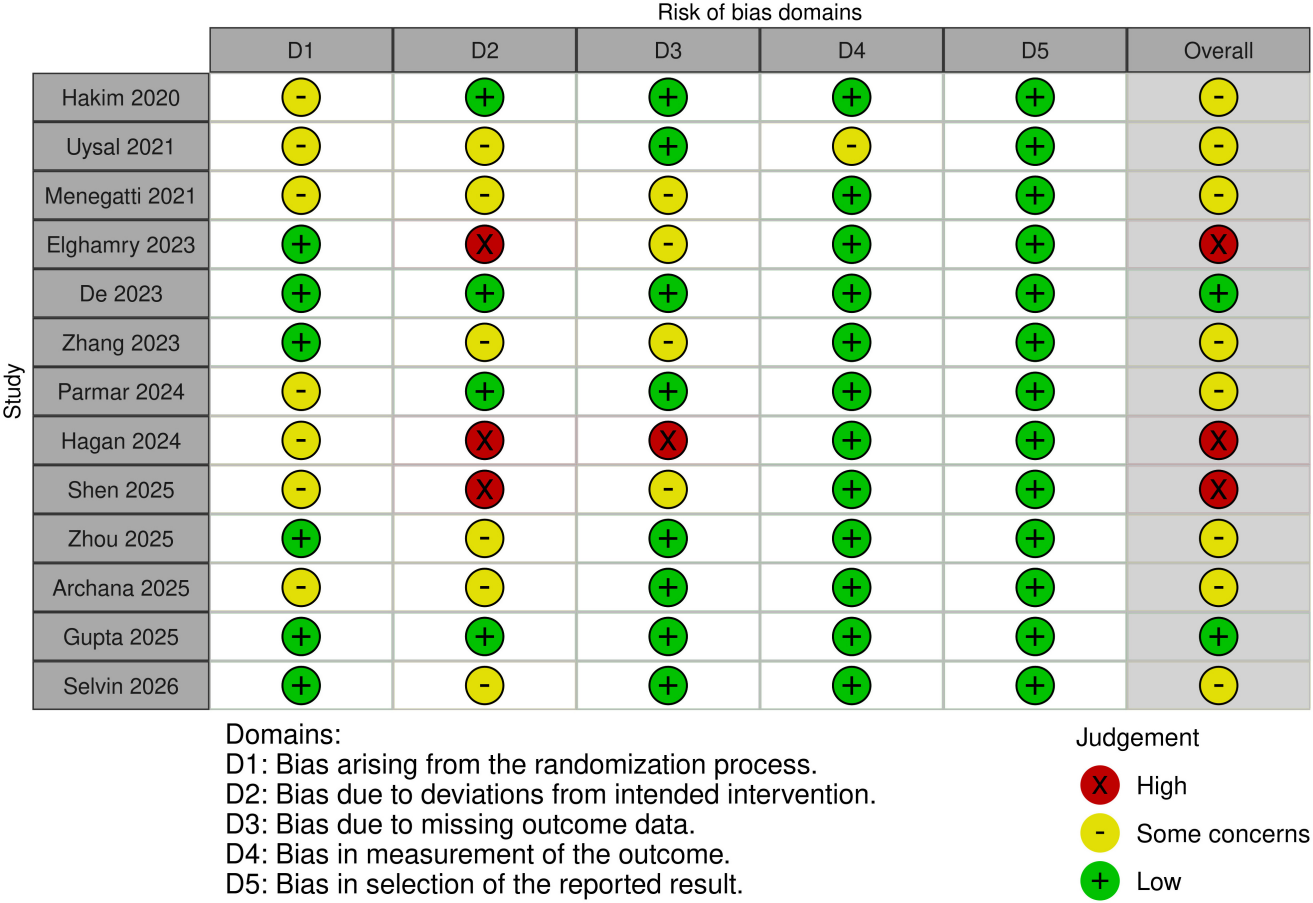
Risk of bias summary for randomized controlled trials included in the quantitative synthesis.

The included studies were published between 2018 and 2026 and were all written in English. Among the 13 RCTs, 5 compared endoscopy-specific dc-SGAs with endotracheal tubes, 4 compared dc-SGAs with other placed airway or access devices, and 4 compared dc-SGAs with non-invasive oxygen therapy. The 2 observational studies consisted of one prospective single-arm observational study and one retrospective observational analysis. The main characteristics of the RCTs are summarized in Table 1. Risk-of-bias assessments for the RCTs are presented in Figure 1.

**TABLE 1:** Characteristics of randomized controlled trials included in the quantitative synthesis.

Study	Country	Design	Sample size	Population	Procedure	Intervention	Comparator	Primary outcome	Remarks
Hakim et al. (16)	USA	RCT	200	Children/adolescents, 5–19 year, ASA I–III	EGD	LMA^®^ Gastro™	Ambu^®^ AuraOnce™ standard LMA	Change in LMA intracuff pressure	Pediatric EGD
Uysal et al. (17)	Turkey	RCT	100	Adults, 18–65 year, ASA I–II	ERCP	LMA^®^ Gastro™	Gastrolaryngeal tube	Oropharyngeal leak pressure	Adult ERCP
Menegatti et al. (26)	Belgium	RCT	65	Adults ≥ 18 year, ASA ≤ III	EGD	LMA^®^ Gastro™	Classic LMA; endoscopy mask	Oxygen saturation during gastroscopy	Three-arm trial; POST timing not predefined
Elghamry et al. (18)	Egypt	RCT	146	Pediatric patients, 8–18 year, ≥ 30 kg, ASA II–III	TEE	LMA^®^ Gastro™	Endotracheal tube	TEE insertion success rate	Pediatric TEE
De et al. (19)	India	RCT	70	Adults ≥ 18 year, ASA I–III, height > 155 cm	ERCP	LMA^®^ Gastro™	Gastrolaryngeal tube	First-time device insertion success rate	Adult ERCP
Zhang et al. (8)	China	RCT	164	Adults with liver cirrhosis undergoing elective EVL; mostly ASA III–IV	EVL	Jcerity Endoscoper	Endotracheal tube	Success rate of EVL procedure	Adult EVL; JEA is similar but not identical to LMA Gastro
Parmar et al. (10)	India	RCT	50	Adults, 18–60 year, ASA I–III	Mixed upper GI endoscopy	LMA^®^ Gastro™	Nasal prongs	Utility of Gastro LMA for airway control and oxygenation	mixed upper GI endoscopy
Hagan et al. (20)	USA	RCT	56	Adults undergoing ERCP, mostly ASA III	ERCP	LMA^®^ Gastro™	Nasal cannula	Desaturation, SpO2 < 90%	Underpowered; 10/36 SGA patients excluded after randomization
Shen et al. (21)	China	RCT	97	Male adults, 50–80 year, ASA I–III, BMI ≤ 28 kg/m^2^	TEE	LMA^®^ Gastro™	Endotracheal tube	Time taken for TEE probe insertion	Adult TEE
Zhou et al. (22)	China	RCT	1255	Adults undergoing upper GI endoscopic treatment under GA	Mixed upper GI endoscopic procedures	Jcerity Endoscoper	Endotracheal tube	First-attempt airway insertion success rate	Largest RCT
Archana et al. (23)	India	RCT	80	Adults, 18–70 year, ASA I–III, elective ERCP	ERCP	LMA^®^ Gastro™	Native airway with nasal cannula	Intraprocedural cardiorespiratory events	No blinding
Gupta et al. (24)	India	RCT	60	Adults, 18–65 year, ASA I–III, therapeutic ERCP under GA	ERCP	LMA^®^ Gastro™	Endotracheal tube	Emergence time and significant respiratory adverse events	ITT analysis
Selvin et al. (25)	India	RCT	138	Adults, 18–60 year, ASA I–II, elective upper GI endoscopy under TIVA	Mixed upper GI endoscopy	LMA^®^ Gastro™	Low-flow nasal cannula	First-attempt endoscope insertion success	2 LMA placement failures excluded

Sample size refers to the number of patients included in the analysis unless otherwise specified. LMA^®^ Gastro™ and Jcerity Endoscoper were classified as endoscopy-specific dual-channel supraglottic airways; however, Jcerity Endoscoper is similar in design concept but not identical to LMA^®^ Gastro™. Studies involving TEE were retained as transesophageal instrumentation evidence and should be interpreted as indirect evidence for gastrointestinal endoscopic procedures. “Mixed upper GI endoscopy” refers to studies including more than one upper gastrointestinal endoscopic procedure, such as diagnostic upper GI endoscopy, ERCP, EVL, biopsy, or other therapeutic endoscopic interventions. ASA, American Society of Anesthesiologists; BMI, body mass index; EGD, esophagogastroduodenoscopy; ERCP, endoscopic retrograde cholangiopancreatography; EVL, endoscopic variceal ligation; GA, general anesthesia; GI, gastrointestinal; ITT, intention-to-treat; JEA, Jcerity Endoscoper Airway; LMA, laryngeal mask airway; POST, postoperative sore throat; RCT, randomized controlled trial; SGA, supraglottic airway; TEE, transesophageal echocardiography; TIVA, total intravenous anesthesia; UGI, upper gastrointestinal.

### 3.2 Airway insertion performance

**FIGURE 2:**
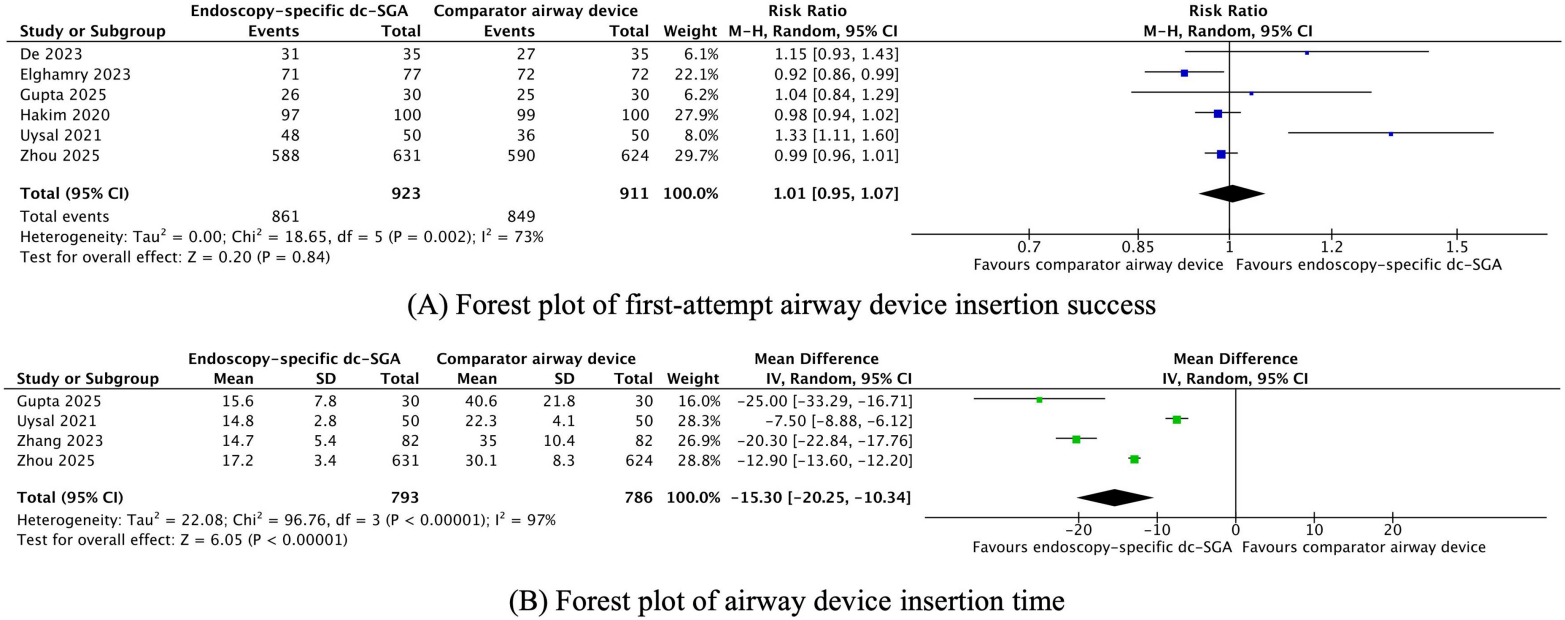
Airway device insertion performance.

Airway device insertion performance is shown in Figure 2. Six trials reported first-attempt airway device insertion success. The pooled analysis showed no significant difference between endoscopy-specific dc-SGAs and comparator airway devices (RR 1.01, 95% CI 0.95–1.07; *P* = 0.84; *I*^2^ = 73%) (Figure 2A). Four trials reported airway device insertion time. Endoscopy-specific dc-SGAs were associated with a significantly shorter insertion time than comparator airway devices (MD -15.30 s, 95% CI -20.25 to -10.34; *P* < 0.00001), although heterogeneity was considerable (*I*^2^ = 97%) (Figure 2B).

### 3.3 Endoscopic/probe insertion performance

**FIGURE 3:**
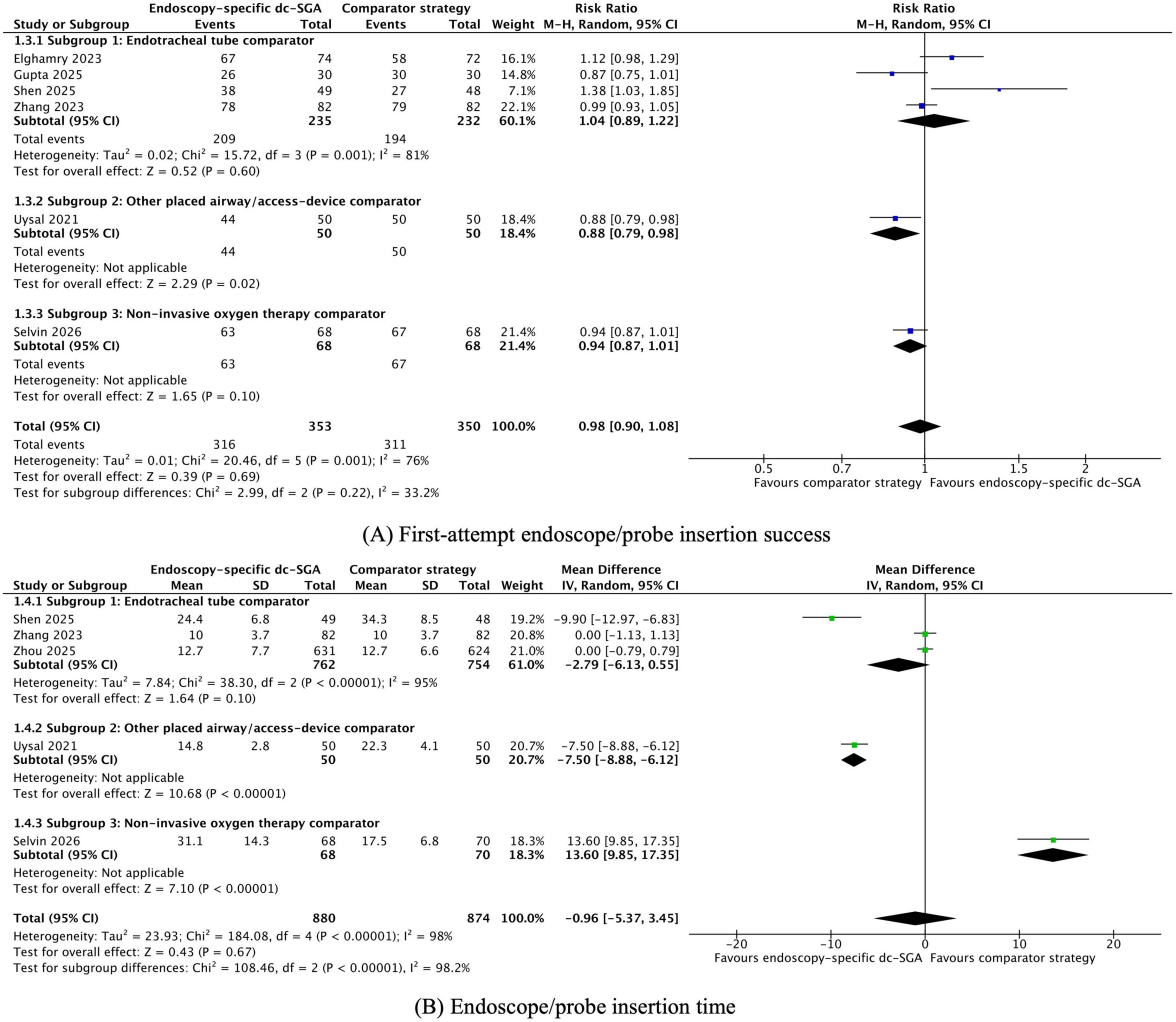
Endoscopic/probe insertion performance.

Endoscopic or transesophageal probe insertion performance is shown in Figure 3. Six trials reported first-attempt endoscope/probe insertion success. The pooled analysis showed no significant difference between endoscopy-specific dc-SGAs and comparator strategies (RR 0.98, 95% CI 0.90–1.08; *P* = 0.69; *I*^2^ = 76%) (Figure 3A). Subgroup analyses showed no significant difference in the endotracheal tube comparator subgroup (RR 1.04, 95% CI 0.89–1.22), while one trial comparing LMA^®^ Gastro™ with a gastrolaryngeal tube favored the comparator device (RR 0.88, 95% CI 0.79–0.98) (Figure 3).

Five trials reported endoscope/probe insertion time. Overall, endoscopy-specific dc-SGAs were not associated with a significant difference in insertion time compared with comparator strategies (MD -0.96 s, 95% CI -5.37 to 3.45; *P* = 0.67; *I*^2^ = 98%) (Figure 3B). The effect varied by comparator type. Compared with endotracheal tubes, dc-SGAs showed no significant reduction in insertion time (MD -2.79 s, 95% CI -6.13 to 0.55). A single trial comparing LMA^®^ Gastro™ with a gastrolaryngeal tube favored dc-SGA (MD -7.50 s, 95% CI -8.88 to -6.12), whereas the trial using non-invasive oxygen therapy as the comparator favored the comparator strategy (MD 13.60 s, 95% CI 9.85–17.35). The test for subgroup differences was significant for insertion time (Figure 3).

### 3.4 Endoscopist satisfaction

**FIGURE 4:**
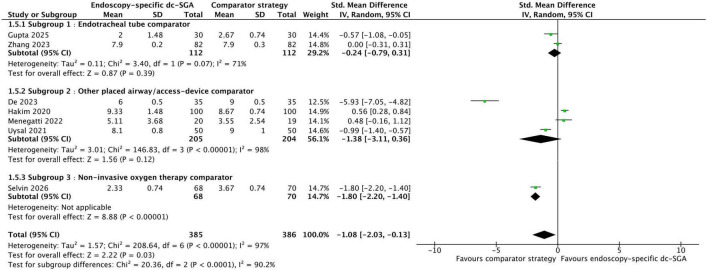
Forest plot comparing endoscopist satisfaction.

Endoscopist satisfaction or procedural ease was reported in seven comparisons. After harmonizing the score direction so that higher scores indicated greater satisfaction or easier procedural conditions, the pooled analysis favored comparator strategies over endoscopy-specific dc-SGAs (SMD -1.08, 95% CI -2.03 to -0.13; *P* = 0.03), with considerable heterogeneity (*I*^2^ = 97%). In subgroup analyses, there was no significant difference in the endotracheal tube comparator subgroup (SMD -0.24, 95% CI -0.79 to 0.31) or in the other placed airway/access-device comparator subgroup (SMD -1.38, 95% CI -3.11 to 0.36). One trial comparing LMA^®^ Gastro™ with non-invasive oxygen therapy favored the comparator strategy (SMD -1.80, 95% CI -2.20 to -1.40). The test for subgroup differences was significant (Figure 4).

### 3.5 Postoperative sore throat

**FIGURE 5:**
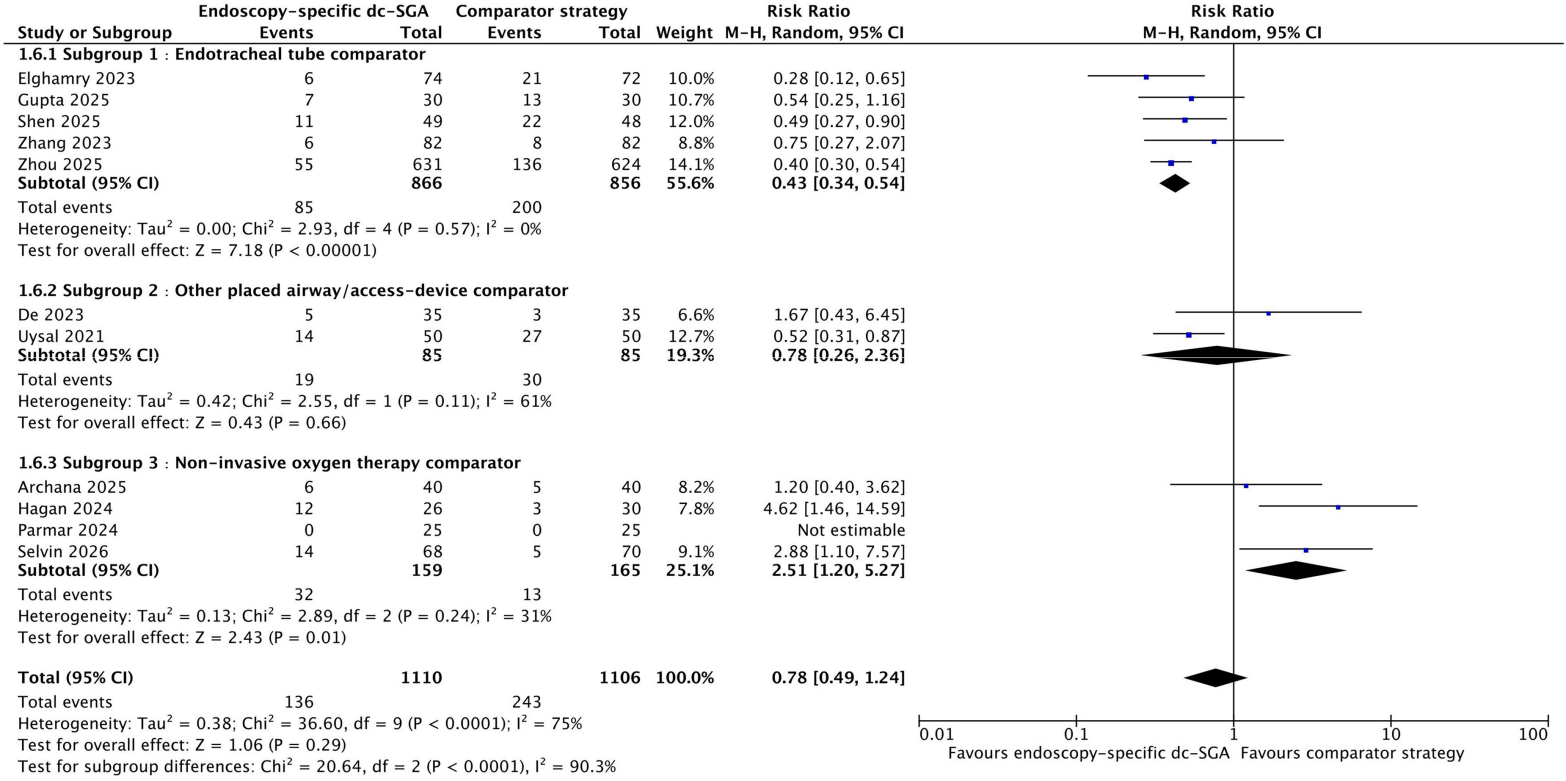
Postoperative sore throat.

Postoperative sore throat was variably assessed across studies, with differences in scoring systems and timing of assessment. Therefore, the primary analysis was restricted to POST incidence, using the earliest postoperative assessment when multiple time points were reported. Overall, endoscopy-specific dc-SGAs were not associated with a significant difference in POST compared with comparator strategies (RR 0.78, 95% CI 0.49–1.24; *P* = 0.29; *I*^2^ = 75%). In subgroup analyses, dc-SGAs were associated with a lower incidence of POST compared with endotracheal tubes (RR 0.43, 95% CI 0.34–0.54; *P* < 0.00001; I^2^ = 0%), but not compared with other placed airway/access devices (RR 0.78, 95% CI 0.26–2.36). In contrast, dc-SGAs were associated with a higher incidence of POST compared with non-invasive oxygen therapy (RR 2.51, 95% CI 1.20–5.27). The test for subgroup differences was significant (Figure 5).

### 3.6 Intraoperative hypoxemia

**FIGURE 6:**
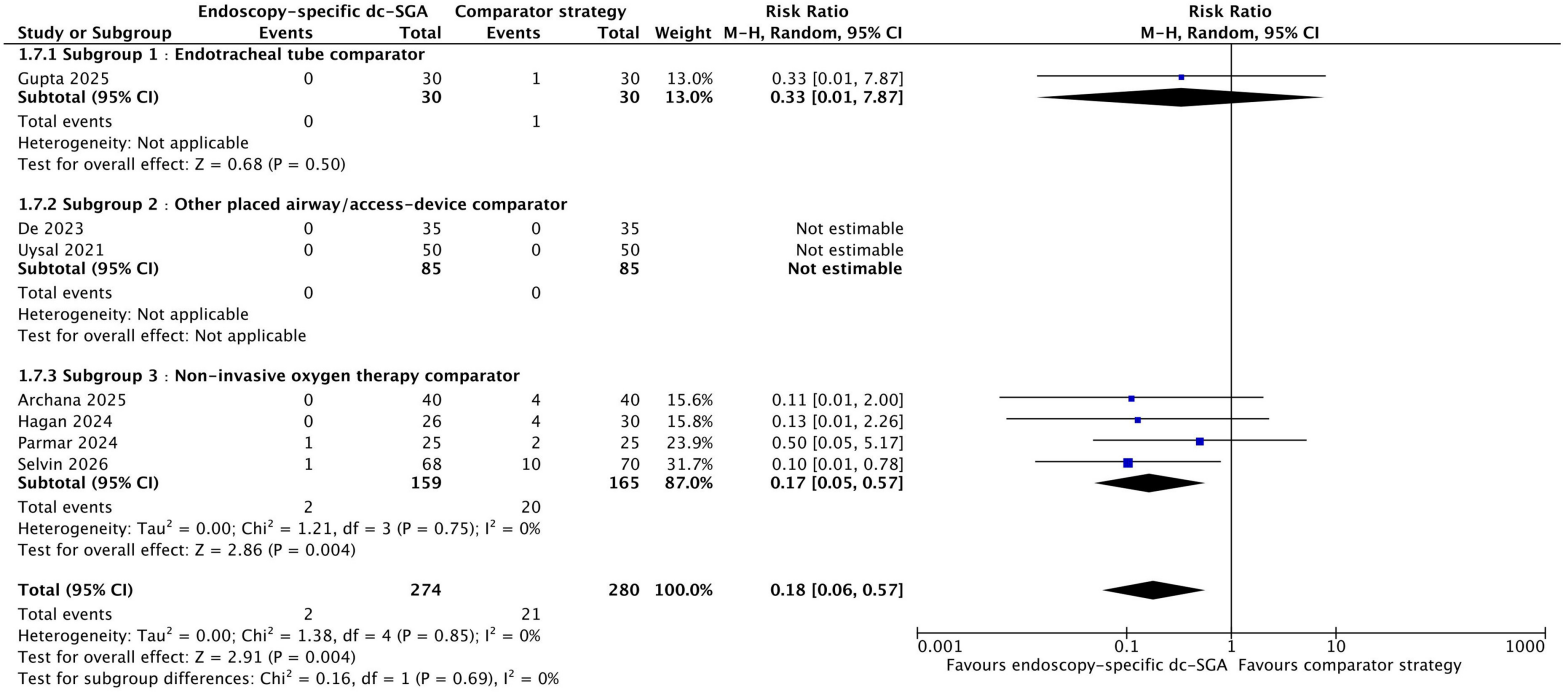
Intraoperative hypoxemia.

Intraoperative hypoxemia was reported in seven trials (Figure 6). Overall, endoscopy-specific dc-SGAs were associated with a lower risk of intraoperative hypoxemia compared with comparator strategies (RR 0.18, 95% CI 0.06–0.57; *P* = 0.004; *I*^2^ = 0%). This finding was mainly driven by trials comparing dc-SGAs with non-invasive oxygen therapy, in which dc-SGAs significantly reduced hypoxemia (RR 0.17, 95% CI 0.05–0.57; *P* = 0.004; *I*^2^ = 0%). In contrast, evidence was limited in comparisons with endotracheal tubes or other placed airway/access devices because hypoxemia events were rare or absent.

Additional outcomes, including blood staining or visible airway trauma, recovery time or recovery-room stay, and postoperative hoarseness, are presented in Supplementary Figures 2–4.

### 3.7 Publication bias and certainty of evidence

Potential publication bias was assessed qualitatively because the number of studies available for most outcomes and comparator-specific subgroup analyses was small. Formal funnel plot assessment and statistical tests for funnel plot asymmetry were not performed, as these methods would be underpowered and potentially misleading with fewer than 10 studies per outcome or subgroup. Therefore, the absence of apparent publication bias should not be inferred, and publication bias could not be reliably excluded.

Using the GRADE approach, the certainty of evidence for the key outcomes ranged from low to moderate. Evidence was downgraded mainly because of inconsistency, imprecision, limited blinding for subjective outcomes, heterogeneity in comparator strategies, variability in outcome definitions, and the limited availability of aspiration-related data. Certainty was moderate for first-attempt airway device insertion success, airway device insertion time, first-attempt endoscope/probe insertion success, postoperative sore throat in comparisons with endotracheal tubes, postoperative sore throat in comparisons with non-invasive oxygen therapy, and intraoperative hypoxemia. Certainty was low for endoscope/probe insertion time, endoscopist satisfaction or procedural ease, and postoperative sore throat in comparisons with other placed airway/access devices. These certainty ratings should be interpreted in the context of clinically heterogeneous procedures, evidence derived largely from selected or specialized centres, and data limited to specific endoscopy-specific dc-SGA devices. Accordingly, the observed effects should be regarded as comparator-specific evidence signals rather than definitive procedure-wide or device-class-wide estimates. A summary of certainty ratings is presented in Table 2.

**TABLE 2:** Summary of certainty of evidence for key outcomes.

Outcome	Effect estimate	Certainty of evidence	Main reasons for rating
First-attempt airway device insertion success	RR 1.01 (95% CI 0.95 to 1.07)	⊕⊕⊕○ Moderate	Objective outcome; downgraded for inconsistency
Airway device insertion time	MD -15.30 s (95% CI -20.25 to -10.34)	⊕⊕⊕○ Moderate	Objective outcome with consistent direction of effect; downgraded for substantial heterogeneity
First-attempt endoscope/probe insertion success	RR 0.98 (95% CI 0.90 to 1.08)	⊕⊕⊕○ Moderate	Objective procedural outcome; downgraded for inconsistency across comparator types
Endoscope/probe insertion time	MD -0.96 s (95% CI -5.37 to 3.45)	⊕⊕○○ Low	Substantial heterogeneity and imprecision
Endoscopist satisfaction or procedural ease	SMD -1.08 (95% CI -2.03 to -0.13)	⊕⊕○○ Low	Subjective outcome, heterogeneous scales, and substantial heterogeneity
Postoperative sore throat: dc-SGA vs. endotracheal tube	RR 0.43 (95% CI 0.34 to 0.54)	⊕⊕⊕○ Moderate	Consistent effect; downgraded for subjective outcome assessment and limited blinding
Postoperative sore throat: dc-SGA vs. other placed airway/access devices	RR 0.78 (95% CI 0.26 to 2.36)	⊕⊕○○ Low	Limited number of studies and imprecision
Postoperative sore throat: dc-SGA vs. non-invasive oxygen therapy	RR 2.51 (95% CI 1.20 to 5.27)	⊕⊕⊕○ Moderate	Clinically plausible direction of effect; downgraded for subjective outcome assessment and variable POST assessment methods
Intraoperative hypoxemia	RR 0.18 (95% CI 0.06 to 0.57)	⊕⊕⊕○ Moderate	Objective outcome with consistent direction of effect; downgraded for variable hypoxemia thresholds and limited event numbers

CI, confidence interval; dc-SGA, dual-channel supraglottic airway; MD, mean difference; POST, postoperative sore throat; RR, risk ratio; SMD, standardized mean difference. Negative MD values indicate shorter time with endoscopy-specific dc-SGAs. For endoscopist satisfaction or procedural ease, score directions were harmonized so that higher scores indicated greater satisfaction or easier procedural conditions. For postoperative sore throat, certainty was assessed separately by comparator type because the direction of effect differed across comparator categories.

## 4 Discussion

### 4.1 Principal findings

In this systematic review and meta-analysis of randomized trials, endoscopy-specific dc-SGAs showed comparable first-attempt airway device and endoscope/probe insertion success to comparator strategies, while shortening airway device insertion time. However, the included comparator strategies were clinically heterogeneous and differed substantially in airway invasiveness, ventilatory support, airway protection, and expected adverse-event profiles. Therefore, the pooled estimates should not be interpreted as a single comparison against a uniform control intervention. Instead, they should be interpreted together with comparator-specific subgroup findings. In particular, dc-SGAs were associated with fewer episodes of intraoperative hypoxemia mainly when compared with non-invasive oxygen therapy, whereas postoperative sore throat showed a comparator-dependent pattern, with fewer events compared with endotracheal tubes but more events compared with non-invasive oxygen therapy. Overall, these findings suggest that endoscopy-specific dc-SGAs may serve as a feasible intermediate airway strategy between non-invasive oxygen therapy and endotracheal intubation in selected clinical contexts. However, given the heterogeneity of procedures, comparator strategies, and device types, these findings should not be interpreted as evidence of universal superiority across all upper gastrointestinal endoscopic or transesophageal instrumentation procedures.

### 4.2 Clinical positioning and aspiration protection

The clinical value of endoscopy-specific dc-SGAs should be interpreted within the broader debate between non-invasive oxygen therapy under deep sedation or monitored anesthesia care and general anesthesia with endotracheal intubation during ERCP and other upper aerodigestive tract procedures (2, 3, 27, 28). Non-invasive oxygen therapy preserves procedural simplicity and may facilitate faster recovery and turnover, but it does not provide a sealed airway or reliable ventilatory support when upper airway obstruction, hypoventilation, or prolonged procedural stimulation occurs (2, 3, 27). Conversely, endotracheal intubation provides the most definitive airway control and facilitates positive-pressure ventilation, but it increases procedural invasiveness and may require deeper anesthesia, neuromuscular blockade, and greater anesthetic resources (2, 27–29). Endoscopy-specific dc-SGAs were developed to address this gap by providing a dedicated ventilation channel while permitting passage of an endoscope or transesophageal probe through a separate procedural channel (6, 9).

A key boundary of this intermediate role is aspiration protection. Airway protection against regurgitation and aspiration remains a major reason for selecting a cuffed endotracheal tube during advanced upper gastrointestinal endoscopic procedures, particularly in patients with active gastrointestinal bleeding, gastric outlet obstruction, severe reflux, delayed gastric emptying, full stomach status, or other high-risk features. Endoscopy-specific dc-SGAs may improve airway patency and ventilatory control, but they do not provide the same definitive tracheal seal as a cuffed endotracheal tube. In the included trials, aspiration and regurgitation were rare, inconsistently reported, and not sufficiently comparable for quantitative synthesis; moreover, many trials enrolled selected patients and often excluded those at high risk of aspiration. Therefore, the limited aspiration-related data should remain central when interpreting the safety profile of endoscopy-specific dc-SGAs. The absence of a clear aspiration signal should not be interpreted as evidence that these devices provide aspiration protection equivalent to a cuffed endotracheal tube. In patients requiring definitive airway protection, endotracheal intubation remains the more appropriate strategy.

### 4.3 Procedural performance and subjective outcomes

The procedural findings suggest that the main advantage of endoscopy-specific dc-SGAs lies in airway establishment rather than in consistently improving endoscope or probe manipulation. This interpretation is consistent with the design rationale of these devices: the airway channel is intended to maintain ventilation, while the separate procedural channel permits passage of an endoscope or transesophageal probe (6, 9). However, successful endoscope or probe advancement depends on factors beyond airway device placement, including procedural-channel alignment, pharyngeal space, patient position, type of instrument, and operator experience. This may explain why dc-SGAs shortened airway device insertion time, but did not consistently improve first-attempt endoscope/probe insertion success, endoscope/probe insertion time, or endoscopist satisfaction. Trials comparing LMA^®^ Gastro™ with gastrolaryngeal tubes or endotracheal tubes also suggest that procedural ease may vary by comparator device and by the specific endoscopic or transesophageal procedure being performed (8, 17, 24). Therefore, the procedural benefit of dc-SGAs should be interpreted primarily as improved airway access and ventilatory workflow, rather than as evidence that these devices necessarily facilitate all aspects of endoscopic or transesophageal probe manipulation.

The trade-off between improved airway control and local airway instrumentation is particularly important when dc-SGAs are compared with non-invasive oxygen therapy. Non-invasive oxygen therapy is simple and avoids direct oropharyngeal device-related stimulation, but it cannot bypass upper airway obstruction or provide reliable positive-pressure ventilation during deep sedation (3, 27). In contrast, endoscopy-specific dc-SGAs provide a dedicated ventilation channel and may reduce hypoxemia by maintaining airway patency during procedures in which the endoscope and airway share the upper aerodigestive tract (10, 20, 23). However, dc-SGAs require oropharyngeal placement and cuff contact, which plausibly explains the higher incidence of postoperative sore throat compared with nasal cannula or nasal prongs. Importantly, POST in this setting should not be attributed solely to the airway device, because endoscope or probe insertion, repeated manipulation, procedure duration, and patient positioning may also contribute to throat discomfort. Because POST definitions, severity thresholds, scoring systems, and assessment time frames varied across studies, pain scores were not pooled and POST was interpreted as a procedure-related symptom rather than a device-specific safety endpoint.

### 4.4 Limitations and future directions

This review has several limitations. Most included trials were single-centre studies with modest sample sizes, and some outcomes were based on few events. Clinical heterogeneity was substantial across procedures, populations, anesthetic techniques, devices, and comparator strategies. Although all included procedures involved upper aerodigestive tract instrumentation competing with airway management, ERCP, diagnostic or therapeutic upper gastrointestinal endoscopy, EVL, and TEE differ in procedure duration, patient positioning, aspiration risk, sedation depth, and airway-management requirements. Procedure-specific subgroup analyses, such as ERCP versus non-ERCP procedures, were considered but not performed because of limited study numbers, mixed-procedure studies, and confounding between procedure type, device type, and comparator strategy. Aspiration-related outcomes were rare, inconsistently defined, and insufficiently reported for quantitative synthesis, limiting conclusions regarding aspiration safety. In addition, the current evidence is largely device-specific and should be interpreted primarily for the LMA^®^ Gastro™ Airway and the Jcerity Endoscoper™ Airway, rather than generalized to all dual-channel supraglottic airways. The predominance of evidence from selected or specialized centres, together with operator experience, learning curves, and institutional familiarity with these devices, may also have influenced airway insertion success, endoscope/probe insertion performance, satisfaction scores, and adverse-event reporting, thereby limiting external validity. Therefore, the pooled estimates should be interpreted as comparative evidence signals rather than procedure-specific or device-class-wide recommendations.

Future trials should move beyond small single-centre comparisons and use adequately powered, multicentre randomized designs with standardized outcome definitions. In particular, future studies should use consistent thresholds for hypoxemia, report the duration and severity of desaturation, and capture clinically important rescue events such as jaw thrust, mask ventilation, airway conversion, procedure interruption, aspiration, and unplanned admission. Because optimized non-invasive oxygen strategies, especially HFNC/HFNO, may reduce hypoxemia during sedated gastrointestinal endoscopy, future studies should compare endoscopy-specific dc-SGAs not only with low-flow nasal cannula oxygen but also with HFNC/HFNO-based protocols (30, 31). Jet ventilation-based approaches, including high-frequency jet ventilation and supraglottic jet oxygenation and ventilation, have also been used as alternative oxygenation or ventilation strategies during ERCP and upper gastrointestinal endoscopy (32). Although these techniques were outside the scope of the present review, future comparative trials should evaluate endoscopy-specific dc-SGAs against jet ventilation-based strategies, HFNC/HFNO, non-invasive oxygen therapy, and endotracheal intubation in clinically relevant patient groups. Dedicated trials in high-risk populations, including patients with obesity, obstructive sleep apnea, advanced cardiopulmonary disease, high ASA physical status, or anticipated difficult airway, are also needed to identify patients most likely to benefit from dc-SGA use (33). Until such evidence is available, endoscopy-specific dc-SGAs should be considered a potentially useful but selective airway strategy: appropriate when improved ventilatory control is desirable, but not a universal replacement for either non-invasive oxygen therapy or endotracheal intubation.

## 5 Conclusion

Endoscopy-specific dc-SGAs may represent a feasible intermediate airway strategy for selected patients undergoing upper gastrointestinal endoscopic and transesophageal instrumentation procedures. Potential benefits in airway placement efficiency and oxygenation should be balanced against comparator-dependent throat discomfort and interpreted cautiously in light of clinical heterogeneity among procedures, limited aspiration-related data, specialized-centre and device-specific evidence, and the low-to-moderate certainty of evidence. Further multicentre randomized trials with standardized outcome definitions are needed.

## Data Availability

The original contributions presented in this study are included in the article/Supplementary material, further inquiries can be directed to the corresponding authors.
