# Supplementary material for: Efficacy and safety of endoscopy-specific dual-channel supraglottic airways for upper gastrointestinal endoscopic and transesophageal instrumentation procedures: a systematic review and meta-analysis
Source: Front Med (Lausanne). 2026 Jul 17;13:1879284. doi: 10.3389/fmed.2026.1879284 (PMC13424288; doi:10.3389/fmed.2026.1879284)
Supplement: Supplementary file 1 [file Supplementary_file_1.docx]

The study selection followed the PRISMA 2020 recommendations. Details of the identification, screening, eligibility assessment, and inclusion of studies are summarized in the PRISMA flow diagram (Figure S1). In brief, 440 records were initially identified from electronic databases and trial registers, and 15 studies finally met the predefined eligibility criteria and were included in the systematic review. Of these, 13 randomized controlled trials were included in the quantitative synthesis, and 2 observational studies were summarized narratively.


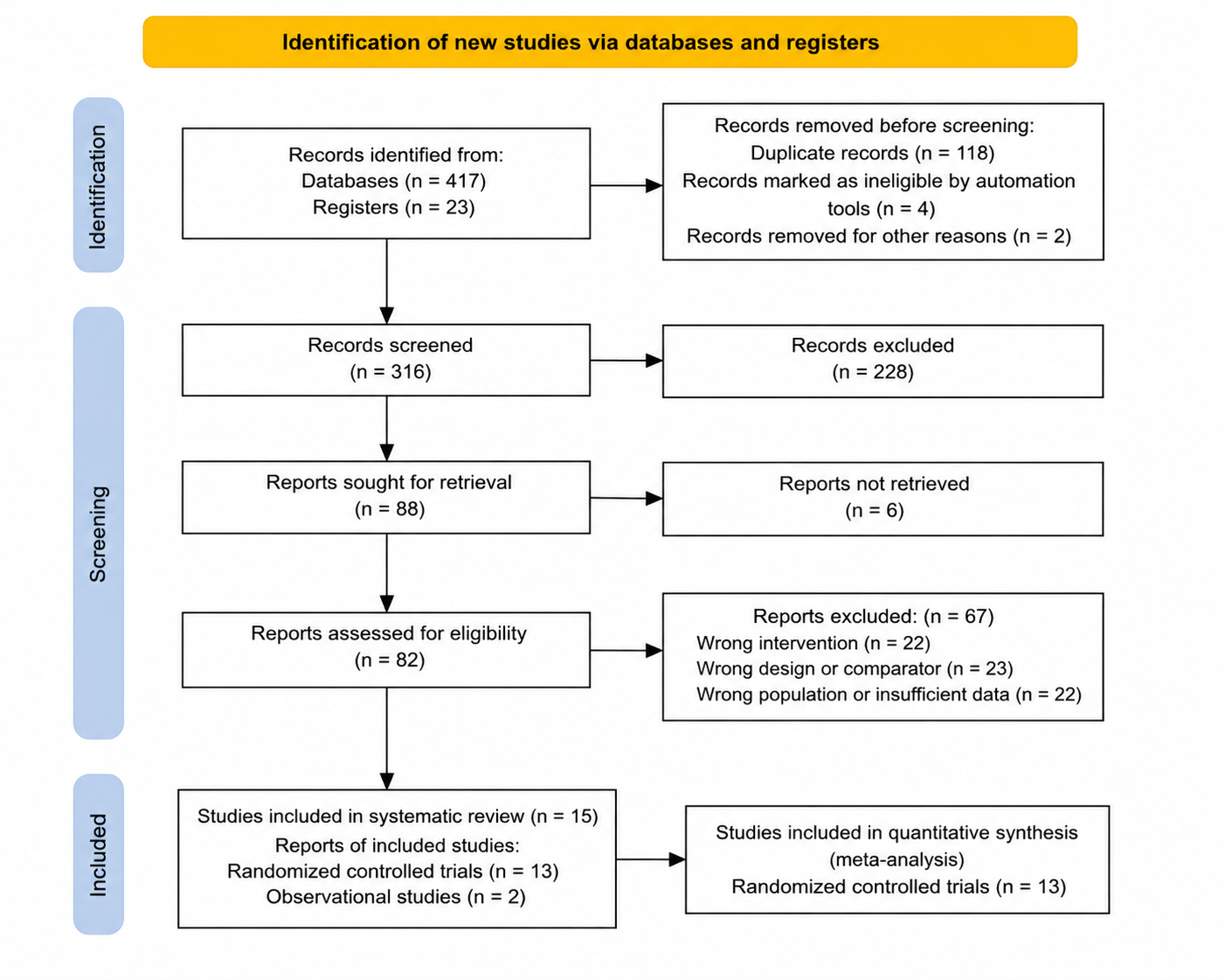


Figure S1. Study selection process according to the PRISMA 2020 statement. A total of 417 records were identified through database searching (PubMed, Embase, Cochrane Library, and Web of Science), and 23 additional records were identified from trial registers. After removal of 112 duplicate records, 4 records automatically marked as ineligible, and 1 record removed for other reasons, 323 records were screened based on title and abstract. Of these, 241 records were excluded as clearly not meeting the inclusion criteria. Eighty-two reports were sought for retrieval, and 5 reports could not be obtained in full text, leaving 77 full-text articles assessed for eligibility. Sixty-two reports were excluded at this stage because of wrong intervention, wrong design or comparator, wrong population, or insufficient data. Ultimately, 15 studies were included in the systematic review, including 13 randomized controlled trials and 2 observational studies. The 13 randomized controlled trials were included in the quantitative synthesis, whereas the 2 observational studies were summarized narratively.
