# Supplementary material for: Efficacy and safety of endoscopy-specific dual-channel supraglottic airways for upper gastrointestinal endoscopic and transesophageal instrumentation procedures: a systematic review and meta-analysis
Source: Front Med (Lausanne). 2026 Jul 17;13:1879284. doi: 10.3389/fmed.2026.1879284 (PMC13424288; doi:10.3389/fmed.2026.1879284)
Supplement: Supplementary file 2 [file Supplementary_file_2.docx]

Supplementary Figure S2：Blood staining or visible airway trauma on airway equipment/device at removal.


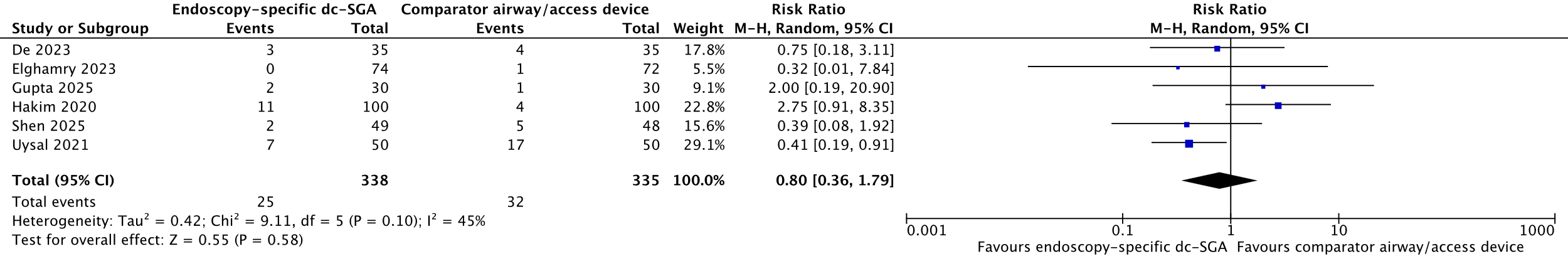


Six studies reported blood staining or visible airway trauma on airway equipment/device at removal. The pooled analysis showed no significant difference between endoscopy-specific dc-SGAs and comparator airway/access devices (RR 0.80, 95% CI 0.36–1.79; P = 0.58), with moderate heterogeneity (I² = 45%). This finding suggests that dc-SGAs were not associated with a clear increase or reduction in visible minor airway trauma compared with other airway/access devices. However, this outcome should be interpreted cautiously because definitions varied across studies and blood staining represents only a surrogate marker of minor upper-airway mucosal irritation rather than a definitive clinically important airway injury endpoint.

Supplementary Figure S3. Recovery time or recovery-room stay.


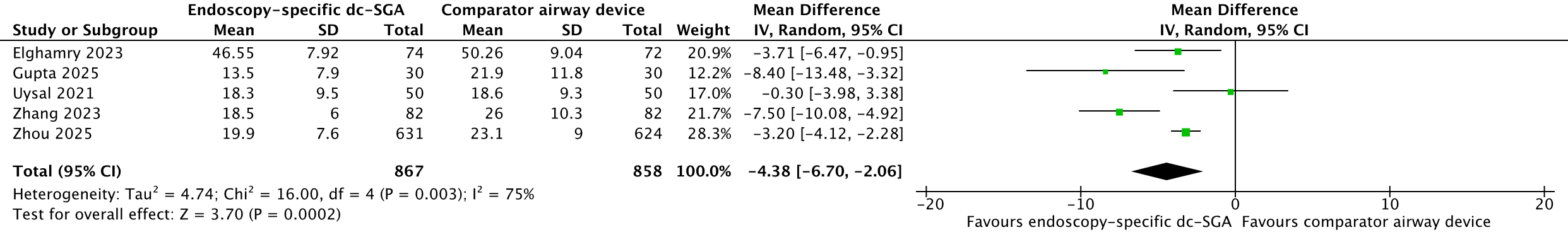


Five studies reported recovery time or recovery-room stay. Endoscopy-specific dc-SGAs were associated with a shorter recovery time or recovery-room stay compared with comparator airway devices (MD −4.38 min, 95% CI −6.70 to −2.06; P = 0.0002). However, heterogeneity was substantial (I² = 75%), likely reflecting differences in recovery definitions, anesthetic protocols, post-anesthesia care unit discharge criteria, and institutional workflow. Therefore, this result should be interpreted as a supportive recovery-related signal rather than a definitive estimate of clinically meaningful time saving.

Supplementary Figure S4. Postoperative hoarseness.


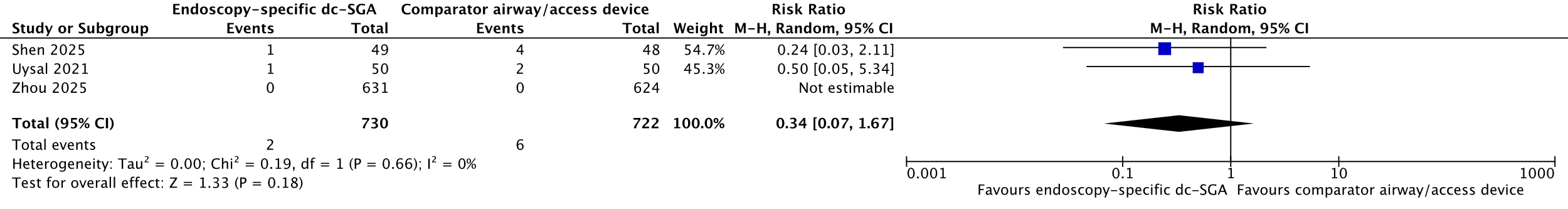
Three studies reported postoperative hoarseness. The pooled analysis showed no significant difference between endoscopy-specific dc-SGAs and comparator airway/access devices (RR 0.34, 95% CI 0.07–1.67; P = 0.18), with no observed heterogeneity (I² = 0%). Although the point estimate favored dc-SGAs, the total number of events was very small and the confidence interval was wide. This outcome should therefore be regarded as an exploratory safety finding rather than evidence of a definite reduction in postoperative hoarseness.
