# Supplementary material for: Efficacy and safety of endoscopy-specific dual-channel supraglottic airways for upper gastrointestinal endoscopic and transesophageal instrumentation procedures: a systematic review and meta-analysis
Source: Front Med (Lausanne). 2026 Jul 17;13:1879284. doi: 10.3389/fmed.2026.1879284 (PMC13424288; doi:10.3389/fmed.2026.1879284)
Supplement: Supplementary file 3 [file Table_1.docx]

**Supplementary Table S1. Search strategies used in the systematic review.**

| Database | Search strategy |
| --- | --- |
| PubMed | (("LMA Gastro"[Title/Abstract] OR "LMA Gastro Airway"[Title/Abstract] OR "LMA GastroTM"[Title/Abstract] OR "LMA® Gastro"[Title/Abstract] OR "Gastro LMA"[Title/Abstract] OR "Gastro laryngeal mask"[Title/Abstract] OR "laryngeal mask airway gastro"[Title/Abstract] OR "dual-channel supraglottic airway"[Title/Abstract] OR "dual channel supraglottic airway"[Title/Abstract] OR "dual-channel laryngeal mask"[Title/Abstract] OR "dual channel laryngeal mask"[Title/Abstract] OR "dual-lumen supraglottic airway"[Title/Abstract] OR "dual lumen supraglottic airway"[Title/Abstract] OR "endoscopy-specific supraglottic airway"[Title/Abstract] OR "endoscopy-specific laryngeal mask"[Title/Abstract] OR "Jcerity Endoscoper"[Title/Abstract] OR "Jcerity Endoscoper Airway"[Title/Abstract] OR "Endoscoper Airway"[Title/Abstract] OR "gastro-laryngeal mask"[Title/Abstract] OR "gastro laryngeal mask"[Title/Abstract]) AND ("endoscopic retrograde cholangiopancreatography"[Title/Abstract] OR ERCP[Title/Abstract] OR "upper gastrointestinal endoscopy"[Title/Abstract] OR "upper GI endoscopy"[Title/Abstract] OR gastroscopy[Title/Abstract] OR oesophagogastroduodenoscopy[Title/Abstract] OR esophagogastroduodenoscopy[Title/Abstract] OR EGD[Title/Abstract] OR OGD[Title/Abstract] OR "endoscopic variceal ligation"[Title/Abstract] OR EVL[Title/Abstract] OR "transesophageal echocardiography"[Title/Abstract] OR "transoesophageal echocardiography"[Title/Abstract] OR TEE[Title/Abstract] OR "transesophageal probe"[Title/Abstract] OR "transoesophageal probe"[Title/Abstract])) |
| Embase | 1. exp laryngeal mask/ OR exp supraglottic airway/ OR exp airway management/ 2. ("LMA Gastro" OR "LMA Gastro Airway" OR "LMA GastroTM" OR "LMA® Gastro" OR "Gastro LMA" OR "Gastro laryngeal mask" OR "laryngeal mask airway gastro" OR "dual-channel supraglottic airway" OR "dual channel supraglottic airway" OR "dual-channel laryngeal mask" OR "dual channel laryngeal mask" OR "dual-lumen supraglottic airway" OR "dual lumen supraglottic airway" OR "endoscopy-specific supraglottic airway" OR "endoscopy-specific laryngeal mask" OR "Jcerity Endoscoper" OR "Jcerity Endoscoper Airway" OR "Endoscoper Airway" OR "gastro-laryngeal mask" OR "gastro laryngeal mask").ti,ab,kw. 3. 1 OR 2 4. exp endoscopic retrograde cholangiopancreatography/ OR exp gastrointestinal endoscopy/ OR exp gastroscopy/ OR exp transesophageal echocardiography/ 5. ("endoscopic retrograde cholangiopancreatography" OR ERCP OR "upper gastrointestinal endoscopy" OR "upper GI endoscopy" OR gastroscopy OR oesophagogastroduodenoscopy OR esophagogastroduodenoscopy OR EGD OR OGD OR "endoscopic variceal ligation" OR EVL OR "transesophageal echocardiography" OR "transoesophageal echocardiography" OR TEE OR "transesophageal probe" OR "transoesophageal probe").ti,ab,kw. 6. 4 OR 5 7. 3 AND 6 |
| Cochrane Library | ("LMA Gastro" OR "LMA Gastro Airway" OR "LMA GastroTM" OR "LMA® Gastro" OR "Gastro LMA" OR "Gastro laryngeal mask" OR "laryngeal mask airway gastro" OR "dual-channel supraglottic airway" OR "dual channel supraglottic airway" OR "dual-channel laryngeal mask" OR "dual channel laryngeal mask" OR "dual-lumen supraglottic airway" OR "dual lumen supraglottic airway" OR "endoscopy-specific supraglottic airway" OR "endoscopy-specific laryngeal mask" OR "Jcerity Endoscoper" OR "Jcerity Endoscoper Airway" OR "Endoscoper Airway" OR "gastro-laryngeal mask" OR "gastro laryngeal mask") AND ("endoscopic retrograde cholangiopancreatography" OR ERCP OR "upper gastrointestinal endoscopy" OR "upper GI endoscopy" OR gastroscopy OR oesophagogastroduodenoscopy OR esophagogastroduodenoscopy OR EGD OR OGD OR "endoscopic variceal ligation" OR EVL OR "transesophageal echocardiography" OR "transoesophageal echocardiography" OR TEE OR "transesophageal probe" OR "transoesophageal probe") |
| Web of Science | TS=(("LMA Gastro" OR "LMA Gastro Airway" OR "LMA GastroTM" OR "LMA® Gastro" OR "Gastro LMA" OR "Gastro laryngeal mask" OR "laryngeal mask airway gastro" OR "dual-channel supraglottic airway" OR "dual channel supraglottic airway" OR "dual-channel laryngeal mask" OR "dual channel laryngeal mask" OR "dual-lumen supraglottic airway" OR "dual lumen supraglottic airway" OR "endoscopy-specific supraglottic airway" OR "endoscopy-specific laryngeal mask" OR "Jcerity Endoscoper" OR "Jcerity Endoscoper Airway" OR "Endoscoper Airway" OR "gastro-laryngeal mask" OR "gastro laryngeal mask") AND ("endoscopic retrograde cholangiopancreatography" OR ERCP OR "upper gastrointestinal endoscopy" OR "upper GI endoscopy" OR gastroscopy OR oesophagogastroduodenoscopy OR esophagogastroduodenoscopy OR EGD OR OGD OR "endoscopic variceal ligation" OR EVL OR "transesophageal echocardiography" OR "transoesophageal echocardiography" OR TEE OR "transesophageal probe" OR "transoesophageal probe")) |
