# Supplementary material for: Efficacy and safety of endoscopy-specific dual-channel supraglottic airways for upper gastrointestinal endoscopic and transesophageal instrumentation procedures: a systematic review and meta-analysis
Source: Front Med (Lausanne). 2026 Jul 17;13:1879284. doi: 10.3389/fmed.2026.1879284 (PMC13424288; doi:10.3389/fmed.2026.1879284)
Supplement: Supplementary file 4 [file Table_2.docx]

Supplementary Table S2 POST assessment methods and time frames

| **Study** | **POST measure** | **Scoring system** | **Time frame** | **Used in meta-analysis** |
| --- | --- | --- | --- | --- |
| Uysal 2021 | Sore throat incidence and severity | Mild/moderate/severe | Early postoperative period | Any POST incidence |
| Elghamry 2023 | Sore throat incidence | Yes/no | Early postoperative period | Any POST incidence |
| Zhang 2023 | Sore throat incidence | Yes/no | Postoperative complications | Any POST incidence |
| Shen 2025/2026 | Sore throat incidence | Yes/no | Before transfer to ward; discharge | Early time point used |
| Zhou 2025 | Pharyngodynia | Yes/no | Postoperative period | Used as POST incidence |
| Gupta 2025 | Sore throat incidence | Yes/no | 30 min and 2 h postoperatively | 30 min used |
| Hagan 2024 | Throat pain | Yes/no | PACU discharge | Sensitivity/narrative |
| Parmar 2024 | Sore throat/hoarseness/dysphagia | Yes/no | Within 4 h | Included as zero-event study if retained |
| Selvin 2026 | Sore throat incidence | Yes/no | Postoperative period | Any POST incidence |
| Hakim 2020 | Throat soreness score | VAS | Before discharge and POD1 | Not pooled as incidence |
| Menegatti 2021 | Sore throat score | VAS | Before and after procedure | Not pooled as incidence |
| Archana 2025 | Moderate-to-severe POST | Severity threshold | Postoperative period | Not pooled with any POST |

Note: POST, postoperative sore throat; PACU, post-anesthesia care unit; POD, postoperative day; VAS, visual analogue scale. Because POST assessment differed across studies in terms of scoring system, severity threshold, and timing, the primary meta-analysis was restricted to POST incidence. When multiple postoperative time points were available, the earliest postoperative assessment was used to avoid within-study duplication. Pain scores, VAS scores, and ordinal severity scales were not pooled because they were not directly comparable across studies. Studies reporting only moderate-to-severe POST were not pooled with studies reporting any POST incidence. POST should be interpreted as a procedure-related throat symptom, because endoscope or probe insertion and procedural manipulation may also contribute to throat discomfort.
