# Supplementary material for: Efficacy and safety of endoscopy-specific dual-channel supraglottic airways for upper gastrointestinal endoscopic and transesophageal instrumentation procedures: a systematic review and meta-analysis
Source: Front Med (Lausanne). 2026 Jul 17;13:1879284. doi: 10.3389/fmed.2026.1879284 (PMC13424288; doi:10.3389/fmed.2026.1879284)
Supplement: Supplementary file 5 [file Table_3.docx]

**Reporting checklist for systematic review (with or without a meta-analysis).**

Based on the PRISMA 2020 guidelines.

**Instructions to authors**

This publishable checklist reports section headings or manuscript locations rather than manuscript page numbers, because page numbers may change after publication.

If an item does not apply, “N/A” is provided with a short explanation.

In the Methods section, the manuscript states that the review was conducted in accordance with the PRISMA 2020 statement and cites the PRISMA 2020 guideline.

Page MJ, McKenzie JE, Bossuyt PM, Boutron I, Hoffmann TC, Mulrow CD, Shamseer L, Tetzlaff JM, Akl EA, Brennan SE, Chou R, Glanville J, Grimshaw JM, Hrobjartsson A, Lalu MM, Li T, Loder EW, Mayo-Wilson E, McDonald S, McGuinness LA, Stewart LA, Thomas J, Tricco AC, Welch VA, Whiting P, Moher D. The PRISMA 2020 statement: an updated guideline for reporting systematic reviews.

| **Reporting item** | **Item** | **PRISMA checklist item** | **Reported on / location** |
| --- | --- | --- | --- |
| **Title** | | | |
| Title | #1 | Identify the report as a systematic review | Title |
| **Abstract** | | | |
| Abstract | #2 | Report an abstract addressing each item in the PRISMA 2020 for Abstracts checklist | Abstract |
| **Introduction** | | | |
| Background/rationale | #3 | Describe the rationale for the review in the context of existing knowledge | Introduction |
| Objectives | #4 | Provide an explicit statement of the objective(s) or question(s) the review addresses | Introduction |
| **Methods** | | | |
| Eligibility criteria | #5 | Specify the inclusion and exclusion criteria for the review and how studies were grouped for the syntheses | Methods: Eligibility criteria |
| Information sources | #6 | Specify all databases, registers, websites, organisations, reference lists, and other sources searched or consulted to identify studies. Specify the date when each source was last searched or consulted | Methods: Search strategy |
| Search strategy | #7 | Present the full search strategies for all databases, registers, and websites, including any filters and limits used | Methods: Search strategy; Supplementary Table S1 |
| Selection process | #8 | Specify the methods used to decide whether a study met the inclusion criteria of the review, including how many reviewers screened each record and each report retrieved, whether they worked independently, and, if applicable, details of automation tools used in the process | Methods: Study selection |
| Data collection process | #9 | Specify the methods used to collect data from reports, including how many reviewers collected data from each report, whether they worked independently, any processes for obtaining or confirming data from study investigators, and, if applicable, details of automation tools used in the process | Methods: Data extraction |
| Data items | #10a | List and define all outcomes for which data were sought. Specify whether all results that were compatible with each outcome domain in each study were sought and, if not, the methods used to decide which results to collect | Methods: Eligibility criteria; Methods: Data extraction |
| Study risk of bias assessment | #11 | Specify the methods used to assess risk of bias in the included studies, including details of the tool(s) used, how many reviewers assessed each study and whether they worked independently | Methods: Risk of bias assessment |
| Effect measures | #12 | Specify for each outcome the effect measure(s), such as risk ratio or mean difference, used in the synthesis or presentation of results | Methods: Statistical analysis |
| Synthesis methods | #13a | Describe the processes used to decide which studies were eligible for each synthesis | Methods: Eligibility criteria; Methods: Statistical analysis |
| Synthesis methods | #13b | Describe any methods required to prepare the data for presentation or synthesis, such as handling of missing summary statistics or data conversions | Methods: Data extraction; Methods: Statistical analysis |
| Synthesis methods | #13c | Describe any methods used to tabulate or visually display results of individual studies and syntheses | Results: Figures 1-6; Supplementary Figures S1-S4; Tables 1-2 |
| Synthesis methods | #13d | Describe any methods used to synthesise results and provide a rationale for the choice(s), including meta-analysis model(s), heterogeneity methods, and software package(s) | Methods: Statistical analysis |
| Synthesis methods | #13e | Describe any methods used to explore possible causes of heterogeneity among study results, such as subgroup analysis or meta-regression | Methods: Statistical analysis; Results: subgroup analyses |
| Synthesis methods | #13f | Describe any sensitivity analyses conducted to assess robustness of the synthesised results | N/A - No sensitivity analyses were conducted. |
| Reporting bias assessment | #14 | Describe any methods used to assess risk of bias due to missing results in a synthesis arising from reporting biases | Results: Publication bias and certainty of evidence |
| Certainty assessment | #15 | Describe any methods used to assess certainty or confidence in the body of evidence for an outcome | Methods: Risk of bias assessment; Results: Publication bias and certainty of evidence; Table 2 |
| Data items | #10b | List and define all other variables for which data were sought, such as participant and intervention characteristics. Describe any assumptions made about missing or unclear information | Methods: Data extraction; Table 1 |
| **Results** | | | |
| Study selection | #16a | Describe the results of the search and selection process, from records identified to studies included, ideally using a flow diagram | Results: Results of literature search; Supplementary Figure S1 |
| Study selection | #16b | Cite studies that might appear to meet the inclusion criteria, but which were excluded, and explain why they were excluded | Results: Results of literature search; Supplementary Figure S1 |
| Study characteristics | #17 | Cite each included study and present its characteristics | Results: Results of literature search; Table 1 |
| Risk of bias in studies | #18 | Present assessments of risk of bias for each included study | Results: Results of literature search; Figure 1 |
| Results of individual studies | #19 | For all outcomes, present for each study summary statistics and effect estimates with precision, ideally using structured tables or plots | Results: Airway insertion performance; Endoscopic/probe insertion performance; Endoscopist satisfaction; Postoperative sore throat; Intraoperative hypoxemia; Figures 2-6; Supplementary Figures S2-S4 |
| Results of syntheses | #20a | For each synthesis, briefly summarise the characteristics and risk of bias among contributing studies | Results: Results of literature search; Figure 1; Table 1; Figures 2-6 |
| Results of syntheses | #20b | Present results of all statistical syntheses, including summary estimates, precision, heterogeneity, and direction of effect | Results: Airway insertion performance; Endoscopic/probe insertion performance; Endoscopist satisfaction; Postoperative sore throat; Intraoperative hypoxemia; Figures 2-6; Supplementary Figures S2-S4 |
| Results of syntheses | #20c | Present results of all investigations of possible causes of heterogeneity among study results | Results: subgroup analyses by comparator strategy; Results: Publication bias and certainty of evidence |
| Results of syntheses | #20d | Present results of all sensitivity analyses conducted to assess robustness of the synthesised results | N/A - No sensitivity analyses were conducted. |
| Risk of reporting biases in syntheses | #21 | Present assessments of risk of bias due to missing results arising from reporting biases for each synthesis assessed | Results: Publication bias and certainty of evidence |
| Certainty of evidence | #22 | Present assessments of certainty or confidence in the body of evidence for each outcome assessed | Results: Publication bias and certainty of evidence; Table 2 |
| **Discussion** | | | |
| Results in context | #23a | Provide a general interpretation of the results in the context of other evidence | Discussion: Principal findings; Clinical positioning and aspiration protection; Procedural performance and subjective outcomes |
| Limitations of included studies | #23b | Discuss any limitations of the evidence included in the review | Discussion: Limitations and future directions |
| Limitations of the review methods | #23c | Discuss any limitations of the review processes used | Discussion: Limitations and future directions |
| Implications | #23d | Discuss implications of the results for practice, policy, and future research | Discussion: Limitations and future directions; Conclusions |
| **Other information** | | | |
| Registration and protocol | #24a | Provide registration information for the review, including register name and registration number, or state that the review was not registered | Title page: Systematic review registration; Abstract: Systematic review registration; Methods: Study design and reporting |
| Registration and protocol | #24b | Indicate where the review protocol can be accessed, or state that a protocol was not prepared | Abstract: Systematic review registration; Methods: Study design and reporting |
| Registration and protocol | #24c | Describe and explain any amendments to information provided at registration or in the protocol | N/A - No amendments to the PROSPERO registration or protocol were reported. |
| Support | #25 | Describe sources of financial or non-financial support for the review and the role of funders or sponsors | Funding |
| Competing interests | #26 | Declare any competing interests of review authors | Conflict of interest |
| Availability of data, code, and other materials | #27 | Report which data, code, and other materials are publicly available and where they can be found | Data Availability Statement; Supplementary material |

The PRISMA checklist is distributed under the terms of the Creative Commons Attribution License CC-BY. This checklist was updated to use section headings/manuscript locations instead of page numbers, as requested by the Editorial Office.
